# Osteocalcin biomarker level evaluation on fracture healing with bone defect after stromal vascular fraction application in murine model

**DOI:** 10.1016/j.amsu.2021.103020

**Published:** 2021-11-02

**Authors:** Respati S. Dradjat, Panji Sananta, Rizqi Daniar Rosandi, Lasa Dhakka Siahaan

**Affiliations:** a Teaching Staff of Orthopaedic and Traumatology Department, Faculty of Medicine Universitas Brawijaya, RSUD Dr. Saiful Anwar, Malang, Indonesia; b Resident of Orthopaedic and Traumatology Department, Faculty of Medicine Universitas Brawijaya, RSUD Dr. Saiful Anwar, Malang, Indonesia; c Research Assistant Orthopaedic and Traumatology Department, Faculty of Medicine Universitas Brawijaya, RSUD Dr. Saiful Anwar, Malang, Indonesia

**Keywords:** *Bone defect*, *Stromal vascular fraction*, *Osteocalcin*

## Abstract

**Introduction:**

Bone defect (3 mm in murine model) is a condition when the bone tissue cannot undergo a natural healing process caused by severe trauma, tumor, or irradiation. A bone defect is a challenge even for experienced Orthopaedic surgeons. Stromal vascular fraction (SVF) is a heterogeneous cell population derived from adipose tissue that results from minimal manipulation of the adipose tissue itself. Several studies have elucidated the effect of either SVF on bone defect healing. However, to the author's knowledge, there is no study evaluating the effect of SVF application on fracture healing, which was measured with osteocalcin biomarker. This study aims to evaluate the effect of SVF application on bone defect healing measured with osteocalcin as a biomarker of bone healing.

**Materials and methods:**

This was an animal study involving twelve Wistar strain *Rattus norvegivus*. They were divided into three groups: negative group (normal rats), positive group (rats with bone defect and treated without SVF application), and SVF group (rats with bone defect and treated with SVF application). After 30 days, the rats were sacrificed, the osteocalcin biomarkers were evaluated. This biomarker was quantified using ELISA.

**Results:**

Osteocalcin biomarker expressions were higher in the group treated with SVF application than those without using SVF. All comparisons of the SVF group and positive control group showed significant differences (p < 0.05).

**Conclusion:**

SVF application could aid the healing process in a murine model with bone defect, marked by increased osteocalcin levels.

## Highlights

• A study evaluating the effect of SVF application on fracture healing, measured with osteocalcin biomarker.
• Osteocalcin is useful in the evaluation of bone turnover and the clinical setting of bone loss.
• SVF could aid the healing process in a murine model with bone defect.
• A bone defect is a challenge even for experienced Orthopaedic surgeons.

## 1 Introduction

A critical bone defect is a condition where the bone is unable to heal itself naturally. A bone defect could happen after major trauma or tumor growth where most bone tissue is lost, or it could happen after irradiation [1]. In humans, it is considered a bone defect when there is a loss of bone components 2–2.5 times the diameter of the bone involved [2], while other researchers define when the distance of the defect is more than 1 cm plus 50% or more the bone circumference [3,4]. In animal research objects such as rats, it is called a bone defect when there is a loss of bone components up to 3 mm [5]. Bone defect is a challenge for orthopaedics, as the condition complicates fracture and needs further reconstruction [6,7].

Stromal vascular fraction (SVF) is a heterogeneous cell population that can be acquired from minimally manipulated adipose tissue. It has been reported that SVF contains various cells like Adipose-derived Mesenchymal stem cells (ADMSC), hematopoietic stem cells (HSC), T regulatory cells (T-reg), and progenitor cells. SVF also contains growth factors such as Insulin-like growth factor-1 (IGF-1), transforming growth factor β (TGF β), and Fibroblast growth factor (FGF), which plays a role in cell proliferation and differentiation [8,9].

The healing process in bone defect goes through several phases: hematoma formation, inflammation, soft callus formation in cartilage, neovascularization, soft callus mineralization, hard callus formation, and remodelling of the osteoclastic hard callus to make flat bones [10,11]. However, this process is not enough to cover critical sized defects in bones. Under the circumstances as such, an autograft is a preferred method to replace bone loss. During bone defect healing, several biomarkers fluctuate dynamically to signify an ongoing bone formation, such as osteocalcin (OC), alkaline phosphatase (ALP) [8]. During a bone formation process, the levels of these biomarkers mentioned above will elevate, thus making them a good parameter for fracture healing with bone defect [12,13].

Osteocalcin, also known as “bone gamma-carboxy glutamic acid (GLA) protein (BGP)," is a noncollagenous protein of the bone matrix. Osteocalcin is a product of differentiated osteoblasts formed by 46–50 amino acids according to the species. Vitamin D directly stimulates osteocalcin's transcription (actually, the gene has a “vitamin D responsive element”), while vitamin K regulates the carboxylation process. In addition, variations of growth factors, hormones, or cytokines can modulate osteocalcin production via signaling pathways or interact with transcription factors acting on the osteocalcin promoter gene. (BGLAP gene on chromosome 1q25-q31.) While extensively transcribed during differentiation from osteoblasts, this gene is generally inactivated. Carboxylation glaucoma is involved in the binding of calcium and hydroxyapatite, allowing deposition of osteocalcin in the mineralized bone matrix [12,14].

The benefit of SVF application in the medical and orthopaedics field have been widely observed. SVF has been used in cases of burn trauma, nerve injury, osteoarthritis, osteonecrosis, rheumatoid arthritis, rupture of Achilles tendon, and growth plate defect [[15], [16], [17]]. A combination of SVF application in bone defect therapy has been done, but there has been no study that measures the effect of SVF application on bone healing from osteocalcin biomarkers. So, in this study, the authors would like to observe the effect of SVF from adipose tissue in the process of bone defect healing, measured by osteocalcin (OC) biomarker.

## 2 Methods and materials

### 2.1 Study design and animal model

This study is an experimental laboratory study with a randomized posttest only control group design. In this study, the parameters measured are the result of the authors' intervention.

The samples in this study are healthy male *Rattus novergicus Wistar strain*, age 12 weeks old, weighs around 250 g with no disabilities in the limbs, and have not been fed any chemical substances. These rats are divided into three groups, with four rats in each group. The groups are further identified as:

(1) Negative group: normal rats without fracture and bone defect and SVF application, (2) positive group: murine model with critical sized fracture and bone defect and without SVF application, (3) SVF group: murine model with critical sized fracture and bone defect and with SVF application. These three groups will be observed for 30 days and tested for osteocalcin biomarker levels. The Ethics Committee of Universitas Brawijaya has approved all animal protocols, and all subsequent experiments were carried out according to the ARRIVE guidelines and regulations [18]. Animals were kept in standard conditions of accredited vivarium, anesthesia and euthanasia were performed under ether anesthesia. Gentle handling, daily cage cleaning and close monitoring were done to minimize animal suffering.

### 2.2 Study procedures

#### 2.2.1 The making of stromal vascular fraction from adipose tissue

Five 12 weeks old male Wistar strain rats were sacrificed by dislocating their cervical. Adipose tissue was harvested from the epididymal and perirenal fat. The rats were in the supine position, and a skin incision was made wide and longitudinal to expose the abdomen. The testicles were removed, and the fat surrounding them was harvested. Perirenal fat was collected by cutting off the innervation from the retroperitoneal fat pad.

The harvested adipose tissue was then washed with a solution of PBS (Phosphate-buffered saline; Sigma-Aldrich, Germany) which contains a mixture of 10% antibiotic-antimycotic, then mashed with a knife. It was then immersed in a 0.075% type IA collagenase mixture (Sigma-Aldrich) and PBS for 30 min at 37 °C. The processed tissue was then strained with a 100 μm mesh (Sigma-Aldrich) and centrifuged om 1200 rpm for 10 min at 20 °C. The supernatant was discarded, and the resulting suspension yielded a heterogeneous cell mixture with an estimate of 2 × 10^6^ cells for 1 g of adipose tissue [19].

#### 2.2.2 Preparation procedure for animal fracture model with bone defect and plaster of paris application

Murine models were acclimatized for 7 days before a bone defect was made in positive and intervention groups. Rats were anaesthetized with 100 mg/kg ketamine injection and intraperitoneal 10 mg/kg xylazine hydrochloride before the procedure. The authors ensured rats were under anesthesia using a pedal reflex technique by extending the extremities and pinching the web between the toes. If murine shied away or twitched a muscle and made a sound, then the anesthesia was not enough. After that, an antibiotic injection of 20 mg/kg Cefazolin was administered on the right leg. The operating area was shaved and cleaned with chlorohezadine. The murine were placed in a prone position on the operating area and incised for 3–4 cm. The incision deepened layer by layer until the bone was exposed. Osteotomy was done using a 3 mm Kerrison, so the bone defect made was 3 mm wide. The intervention was then done according to the assigned groups. Then Plaster of Paris was applied from the proximal femur to the ankle with a 90° flexion on the knee. Analgesia was given every 8 h (using IM 5 mg/kg Ketorolac) and an antibiotic was administered 24 h post-surgery using intramuscular 20 mg/kg cefazoline. Monitoring was done periodically for 30 days.

#### 2.2.3 Laboratory analysis with ELISA method

After 30 days, murine models were harvested. The area of bone defect with callus formation was collected and then extracted. The levels of osteocalcin were assessed using the ELISA method.

### 2.3 Statistical analysis

Statistical Package for the Social Sciences (SPSS) was used for statistical analysis purpose and study data are available for access. The hypothetical comparative test steps are as follows: data normality test, variant homogeneity test, and comparative student T-test or One-way ANOVA test or Kruskal-Wallis test according to the normality and homogeneity test result. If ANOVA or Kruskal-Wallis test result is significant (p < 0.05), then the next test is post hoc test. If the data collected was not homogenous by ANOVA, a non-parametric test with the Kruskall-Wallis method can be done.

## 3 Results

The osteocalcin (OC) level was measured using the One-Way ANOVA test because the data were normally distributed and homogenous. The results are depicted in Table 1.

**Table 1:** The Comparison of Osteocalcin Level using One-Way ANOVA Test.

Group	Mean ± SD		p-value
Negative	24.7545 ± 2.8398	a	0.008
Positive	25.157 ± 3.10199	a	
SVF	35.8458 ± 1.61377	b	

Note: Different letter signifies significant difference (p < 0.05) and vice versa.

ANOVA test yielded p-value of 0.008, less than α = 0,05 (p < 0,05). Thus, it is concluded that SVF application has a significant effect on increasing the production of osteocalcin (OC). After that, the test continues the post hoc test (Table 2) to know how the differentiation on each intervention. The result is that the group that received SVF application is significantly different (p < 0,05) compared with the group without SVF.

**Table 2:** Post hoc testing effect of SVF application on osteocalcin levels.

Comparison of Groups		p-value	Note
Negative	Positive	0.974	Not significantly different
	SVF	0.001	Significantly different
Positive	Negative	0.974	Not significantly different
	SVF	0.001	Significantly different
SVF	Negative	0.001	Significantly different
	Positive	0.001	Significantly different

**Fig. 1:**
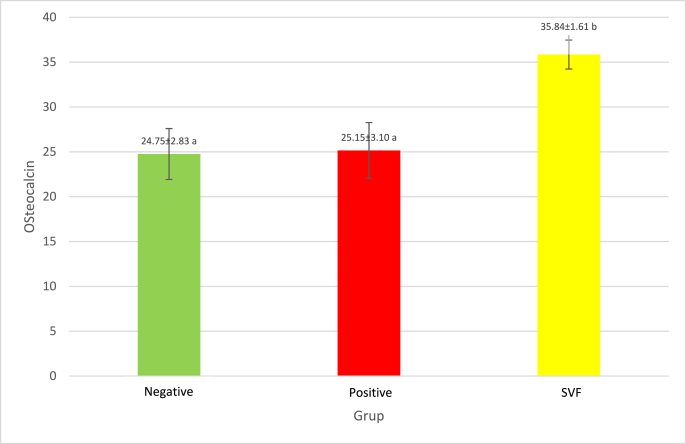
Mean expression of *Osteocalcin* (ng/ml) according to different intervention.

The mean expression of osteocalcin (OC) in each group is depicted in Fig. 1.

## 4 Discussion

This study was an in vivo experimental study on a rat fracture model with a bone defect that received interventions with SVF application. An in vitro study of SVF by Sananta (2020) [20] showed that the characteristic phenotypes of MSC yield positive for CD44 and negative for CD45. This statement aligned with previous findings that if SVF contains MSCs. One of the prerequisite criteria that define MSC is the expression of a specific surface antigen, in this case is CD44, which is a specific characteristic of MSC. A negative finding for CD45 showed that the stem cell originates from adipose tissue instead of hematopoietic cells [21].

SVF of adipose tissue is a heterogeneous population of cells derived from adipose tissue obtained from minimal manipulation of the adipose tissue. The SVF contains a heterogeneous collection of cells and several components, primarily: MSCs, HSCs, Treg cells, pericytic cells, AST cells, complex microvascular beds (fibroblasts, white blood cells, dendritic cells, intra-adventitial smooth muscular-like cells), and the extracellular matrix. In addition, SVF also contains many growth factors, such as Vascular Endothelial Growth Factor (VEGF), Hepatocyte Growth Factor (HGF), IGF-1, TGFβ, and Basic Fibroblast Growth Factor (bFGF). ADMSCs contained in the stromal vascular fraction can continue to produce growth factors [20,21].

SVF will provide sufficient stem cells and growth factor content, especially TGF-beta and BMP-2, provided in SVF. The growth factor will initiate chemoattractant stem cells (mesenchymal cells) to the site of bone defects, then simultaneously increase the proliferation of stem cells and the differentiation of mesenchymal cells into osteoblasts. Where this osteoblast will perform its role in the bone healing process [20].

Osteocalcin is a 49-amino acid protein, which is produced by osteoblasts during bone formation. Osteocalcin is trapped in the bone matrix or released directly into the blood circulation. Osteocalcin was initially studied as a marker of bone formation, with peak levels observed in adolescence and decreasing along with age in both genders. This protein is useful in the evaluation of bone turnover and the clinical setting of bone loss [14].

In this study, there was a significant difference in the levels of osteocalcin after application of SVF (p = 0.008) compared to the control (positive) group and confirmed with post hoc test, which also shows that there is a significantly different effect from intervention (SVF) group to positive group. That implies that the SVF application will increase the level of osteocalcin in fractures with bone defects because osteocalcin is produced by osteoblasts, so an increase in osteocalcin level indicates an increase in osteoblasts activity at the time of bone healing.

From this study, it can be concluded that the application of SVF could aid the healing process in a murine model with bone defect, marked by increased levels of osteocalcin as a bone formation marker. We suggest further study to use a combination of SVF and scaffold and use a different fixation such as an external fixation or an internal fixation to expand the study towards various modalities used in orthopaedics fields. Different biomarkers could also be assessed in future studies, such as ALP, osteopontin, type II collagen, and others, as well as from a histological standpoint.

## Ethical approval

The Ethics Committee of Universitas Brawijaya has approved all animal protocols, and all subsequent experiments were carried out according to the relevant guidelines and regulations.

## Provenance and peer review

Not commissioned, externally peer reviewed.

## Funding

This research did not receive any specific grant from funding agencies in the public, commercial, or not-for-profit sectors.

## Author contribution

Study Design: Respati S Dradjat, Panji Sananta, Rizqi Daniar Rosandi, Lasa Dhakka Siahaan; Data Collection: Respati S Dradjat, Panji Sananta, Rizqi Daniar Rosandi; Statistical Analysis: Respati S Dradjat, Panji Sananta, Rizqi Daniar Rosandi, Lasa Dhakka Siahaan; Data Interpretation: Respati S Dradjat, Panji Sananta, Rizqi Daniar Rosandi, Lasa Dhakka Siahaan; Manuscript Preparation: Respati S Dradjat, Panji Sananta, Rizqi Daniar Rosandi, Lasa Dhakka Siahaan; Literature Search: Panji Sananta, Rizqi Daniar Rosandi, Lasa Dhakka Siahaan.

## Declaration of competing interest

None.
